# Trichotillomania in adulthood, a case report

**DOI:** 10.1192/j.eurpsy.2022.469

**Published:** 2022-09-01

**Authors:** L. Navarro, T. Fernández, L. Tardon, O. Marco, N. Arbelo, N. Baldaquí, M. Cavero

**Affiliations:** 1 HOSPITAL CLÍNIC DE BARCELONA, Psychiatry, BARCELONA, Spain; 2 Hospital Clínic Barcelona, Psychiatry, Barcelona, Spain; 3 Hospital Clínic de Barcelona, Consultation Liaison Psychiatry, Barcelona, Spain; 4 Hospital Clínic, department Of Psychiatry And Psychology, Barcelona, Spain

**Keywords:** Trichotillomania

## Abstract

**Introduction:**

Trichotillomania is a disorder (estimated prevalence 0.5-2.0%) with common onset in childhood, rarely seen in adulthood, characterized by the repetitive pulling out of one’s own hair leading to hair loss and functional impairment, associated with other comorbidities: major depression (39-65%), anxiety disorder (23-32%), SUDs (15-19%), OCD (13-27%).

**Objectives:**

To present a case of late-onset trichotillomania in a 60-year-old woman.

**Methods:**

The present study is a case report of a patient visited in outpatient psychiatry for trichotillomania. We also searched previously case reports, series and systematic reviews of clinical trichotillomania using a pubmed query.

**Results:**

This is a 60-year-old morbidly obese woman diagnosed with dysthymia, binge eating disorder and histrionic personality disorder. She explained a worsening of anxiety associated with work problems of one year of evolution and, for six months, the beginning of the plucking of eyebrow hairs and scabs to decrease this symptom, with inability to avoid the behaviour and without eating the hairs. The mental evaluation highlighted psychic anxiety, hypothymia, low self-esteem and feelings of failure and did not suggest a delirium. We started treatment with topiramate up to 150mg/day which was not successful. After that we switched to fluoxetine up to 60mg/day associated to psychotherapy observing a slight gradual improvement.

**Conclusions:**

The clinical presentation suggested the diagnosis of trichotillomania in the context of dysthymia. No particular medication demonstrates efficacy in the treatment of trichotillomania. Preliminary evidence suggests treatment effects of clomipramine, NAC and olanzapine based on individual trials with small sample sizes. Research findings also recommend psychotherapy based on habit reversal.

**Disclosure:**

No significant relationships.

